# Artificial Intelligence for Image-Based Breast Cancer Risk Prediction Using Attention

**DOI:** 10.3390/tomography9060165

**Published:** 2023-11-24

**Authors:** Stepan Romanov, Sacha Howell, Elaine Harkness, Megan Bydder, D. Gareth Evans, Steven Squires, Martin Fergie, Sue Astley

**Affiliations:** 1 Division of Informatics, Imaging and Data Science, University of Manchester, Manchester M13 9PT, UK; elaine.f.harkness@manchester.ac.uk (E.H.); martin.fergie@manchester.ac.uk (M.F.); 2 Division of Cancer Sciences, University of Manchester, Manchester M20 4GJ, UK; sacha.howell@nhs.net; 3 Department of Medical Oncology, The Christie NHS Foundation Trust, Manchester M20 4BX, UK; 4 The Nightingale Centre, Manchester University NHS Foundation Trust, Manchester M23 9LT, UK; megan.bydder@mft.nhs.uk (M.B.); gareth.d.evans@manchester.ac.uk (D.G.E.); 5 Division of Evolution, Infection and Genomics, University of Manchester, Manchester M13 9PT, UK; 6 Department of Clinical and Biomedical Sciences, University of Exeter, Exeter EX4 4PY, UK; stevensquires@gmail.com

**Keywords:** mammography, multiple instance learning, risk prediction, deep learning, attention

## Abstract

Accurate prediction of individual breast cancer risk paves the way for personalised prevention and early detection. The incorporation of genetic information and breast density has been shown to improve predictions for existing models, but detailed image-based features are yet to be included despite correlating with risk. Complex information can be extracted from mammograms using deep-learning algorithms, however, this is a challenging area of research, partly due to the lack of data within the field, and partly due to the computational burden. We propose an attention-based Multiple Instance Learning (MIL) model that can make accurate, short-term risk predictions from mammograms taken prior to the detection of cancer at full resolution. Current screen-detected cancers are mixed in with priors during model development to promote the detection of features associated with risk specifically and features associated with cancer formation, in addition to alleviating data scarcity issues. MAI-risk achieves an AUC of 0.747 [0.711, 0.783] in cancer-free screening mammograms of women who went on to develop a screen-detected or interval cancer between 5 and 55 months, outperforming both IBIS (AUC 0.594 [0.557, 0.633]) and VAS (AUC 0.649 [0.614, 0.683]) alone when accounting for established clinical risk factors.

## 1. Introduction

Breast cancer is the most commonly diagnosed cancer worldwide. Various factors lead to the increased incidence, with lifestyle, environment, and wider implementation of breast screening all playing a role [1]. Meanwhile, continued improvements in breast cancer treatment and early detection have resulted in mortality decreasing [2].

Screening programmes are designed to screen populations most at risk from cancer and detect malignancies early. The UK National Screening Committee recommends triennial mammography screening starting at 50 years of age until 70 [3], however, guidelines differ between countries [4]. Despite their success, the lack of personalised screening creates several issues including overdiagnosis and differential uptake amongst different groups of women [5]. However, before personalised screening can even be considered, we need to provide clinicians with better, more custom breast cancer risk prediction tools.

The currently used breast cancer risk models primarily use a mix of clinical factors, family history information, and a limited amount of image-based data. Models like the Tyrer-Cuzick (IBIS) [6,7] work well on large populations but leave room for improvement when it comes to individual risk or short-term risk. This is demonstrated by their high calibration but moderate discrimination accuracy [8,9]. However, recent research [10] has started placing increased importance on a broader range of mammographic risk factors, which are normally underrepresented in classic risk models and can be difficult to incorporate into models like IBIS without placing a burden on radiologists. Integrating mammographic features into risk models can encompass a wide range of breast disease markers, including patterns of dense tissue [11], the presence of microcalcifications [12], and other subtle features associated with breast cancer risk, independently of breast density alone.

Hence, our primary objective is to build an accurate, image-based breast cancer risk model, with a specific focus on short-term risk. In particular, by focusing on mammograms taken between 5 and 55 months prior to the detection of breast cancer, our models may be more clinically useful, as it would allow us to adjust individual screening protocols based on model outputs in real time. This range generally encompasses the first prior screening mammogram according to the UK breast screening programme. Our model has the promise of further reducing the rates of interval cancer and improving early diagnosis.

Deep learning models based on Convolutional Neural Networks (CNN) are an attractive solution due to their success in various medical fields. Despite their inclination for processing complex image-based information, their implementation poses several challenges. Mammography images are highly dimensional and most CNNs require a significant amount of computational power. Typically, this issue is resolved by downsizing, but in the field of risk prediction, downsizing can lead to loss of fine information such as calcifications which play an important role in risk prediction [12]. Additionally, models require an adequate amount of labeled data in order to achieve good performance. This is a more challenging issue for risk prediction however as it requires adequate follow-up to determine outcomes, limiting the quantity of data available.

Inspired by the recent success of Multiple Instance Learning (MIL) approaches for histopathology, we propose the Manchester Artificial Intelligence risk model (MAI-risk), our image-based breast cancer risk prediction tool. Our aim is to solve the aforementioned issues without compromising image resolution or computational viability. Using MIL, smaller sections of a given mammogram can be systematically analysed for signs of breast cancer risk preserving small features that may disappear during downsizing. MIL approaches have already been successfully used for image-based breast cancer detection problems. Shen et al. [13] used a mixed global and local network to detect malignant lesions. Other methods [14,15] have also been employed. What they all share in common is the use of MIL to find a malignant lesion—a specific artifact contained in a small region of the breast. What separates risk prediction from these approaches is the higher number of potential features correlating with risk that need to be investigated with none of said features being solely responsible for the decision-making process.

Given our goal of short-term risk prediction specifically, we placed particular emphasis on the data used in the development of our models. The distinction between early cancer detection and accurate risk predictions can sometimes become unclear, which is why our training data incorporates screening mammograms from women who were diagnosed with breast cancer at the time and mammograms from women confirmed cancer-free, but who then went on to develop breast cancer in the future. By mixing cancer-free priors and screen-detected cancers, our aim is to ensure the model can identify mammographic features that correlate with breast cancer risk and features that signify early cancer gestation itself.

## 2. Materials and Methods

### 2.1. Data

Our method uses subsets of the mammograms from the Predicting Risk of Cancer At Screening (PROCAS) study [16]. We extracted screening mammograms from a total of 5064 women, case-control matched, with 1266 cases and 3798 controls. Exclusion criteria included: women with missing Full-Field Digital Mammography (FFDM), women with breast implants in at least one breast, women with missing follow-up, and women with a previous breast cancer diagnosis. The follow-up period was determined by the date of the screening which results in a positive cancer diagnosis for cases. For controls, the last known date of a negative breast screen or the date of death was used. This set is referred to as PROCAS Remaining Cancer mixed or PRC-mixed.

The data is matched with a positive-to-negative ratio of 1 to 3 on age (± 3 years), Body Mass Index (BMI) group (<25 kg/m${}^{2}$, 25–30 kg/m${}^{2}$, >30 kg/m${}^{2}$), parity (Yes/No), Hormone Replacement Therapy (HRT) use (never/ever), and menopausal status (premenopausal/perimenopausal/postmenopausal) in order to account for the variables most associated with risk. Given that the PROCAS study was conducted over a period spanning several years, from 2009 to 2015, the women were additionally matched on year of entry to adjust for potential changes in imaging technology and screening practice.

Of the 1266 cases, 400 are considered biopsy-proven screen-detected cancers, 602 are priors of screen-detected cancers where a woman was confirmed cancer-free at the time of the mammogram but had a subsequent screen-detected cancer up to 9 years in the future, and 264 are priors of future interval cancers where a woman was confirmed cancer-free at the time of the mammogram but had a subsequent interval cancer up to 12 years in the future.

Testing is performed on an independent set of screening mammograms from 1280 women of which 320 were read as cancer-free but the women developed breast cancer between 5 and 55 months later, matched with mammograms of women who remained cancer-free. The testing set had identical matching variables to the train set but had stricter matching criteria for age: ±1 year. This is due to an insufficient number of matching candidate controls for the train set. This set is identical to those used in previous studies from our lab to ensure an even comparison [16] (Study 2). This set is referred to as Breast Cancer Research priors or BCR-priors. The precise composition of both of these sets is provided in Appendix A Table A1.

Additionally, for those women who went on to develop breast cancer, clinical follow-up was used to determine the cancer group, which refers to the type of screening mammogram based on follow-up. The group breakdown is as follows:

- Screen-Detected Cancers (SDC): women who were diagnosed with screen-detected breast cancer on study entry;
- Future Screen-Detected Cancers 1/2/3 (FSDC): women who were confirmed cancer-free on study entry but went on to develop screen-detected cancer. Numbers refer to which subsequent screen was used, so FSDC 1 refers to women who were diagnosed on the first subsequent screen and so on;
- Interval 1/2/3: women who were confirmed cancer-free on study entry but went on to develop an interval cancer after entry. The number refers to before which subsequent screen was the interval diagnosed, so Interval 1 refers to women who developed an interval cancer before the first subsequent screen.

**Figure 1:**
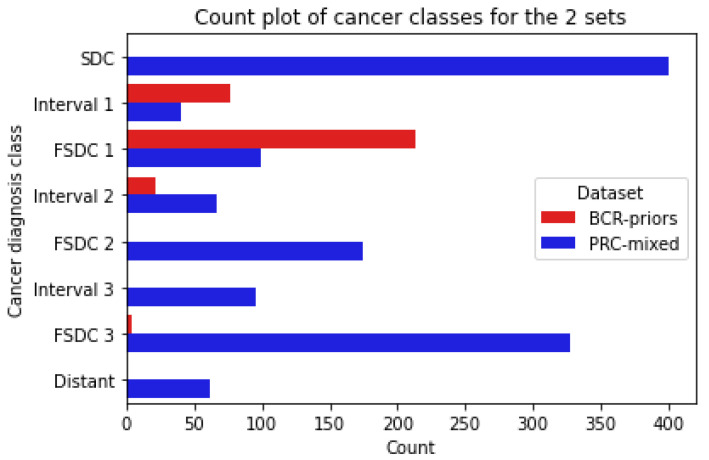
Count plot displaying the cancer group compositions in our datasets.

The dataset cancer group composition can be seen in Figure 1 and outlines the types of cancers we have available, as well as the relative time between study entry and cancer diagnosis for cases. We are considering this approach in order to investigate whether model performance suffers when trying to predict distant cancers and establish the skew of our model towards early detection rather than true risk prediction.

### 2.2. Data Preparation

Despite all 6330 women across both sets having both the raw (for processing) and processed mammograms available, only the latter were used based on experimental results. For cases, only the side that develops cancer was used whilst the other side was removed. For controls, one side was selected randomly and the other was removed. Images had one of two possible dimensions, 3062 × 2394 or 2294 × 1914. Despite the different sizes, the pixel ratios were consistent between them meaning that all patches captured the same relative area regardless of original resolution.

Each image was first max-min standardized, and then Otsu’s segmentation [17] was applied to remove non-zero values from the background. All right-side mammograms were flipped horizontally. Subtle affine transforms were applied during training which included: rotations $\left(-10^{\circ },10^{\circ }\right)$, translations (−100,100 pixels), scaling (0.9,1.1) and shear ${\left(-10^{\circ },10^{\circ }\right)}^{2}$ to improve generalisability. The images were then normalized using our dataset mean and standard deviation of *u* = 0.567 and sd = 0.209.

**Figure 2:**
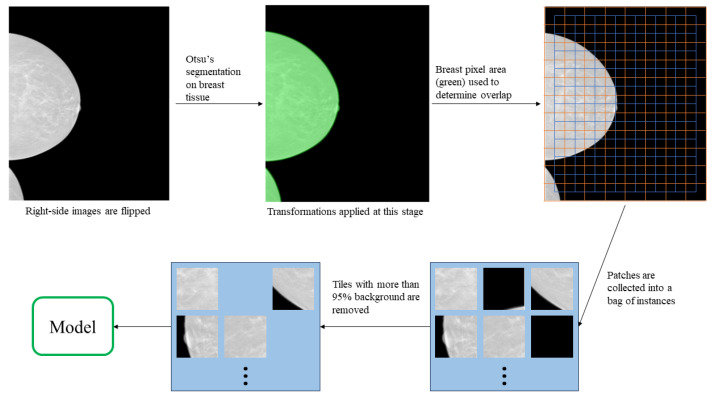
The data pre-processing description. Otsu’s segmentation is applied to detect the breast tissue area and homogenise the background. During training, random transformations are applied at this stage. Next, the total breast pixel area is used to determine the step size for the patching process (step size is 112 in this image). Finally, patches with more than 95% background are removed from the bag.

Images were then patched into smaller, equally sized 224 × 224 tiles with overlap producing a bag of instances. This tile size was selected in order to be compatible with modern CNN networks. Any patch that contained more than 95% background by pixel area was removed due to not capturing enough breast tissue. Each tile was replicated three times across the channel dimension in order to be compatible with modern CNN architectures. The process is represented visually in Figure 2. Women with different breast sizes generated a varying quantity of tiles, which was adjusted using the overlap step size parameter. This is outlined in Table 1.

An identical overall procedure was used for both the craniocaudal (CC) and mediolateral oblique (MLO) views for every woman. One of the advantages of our approach is that by patching the mammogram into smaller areas of breast tissue, the major differences between the shape of CC and MLO mammograms are eliminated and the same model may be used.

### 2.3. Model

**Figure 3:**
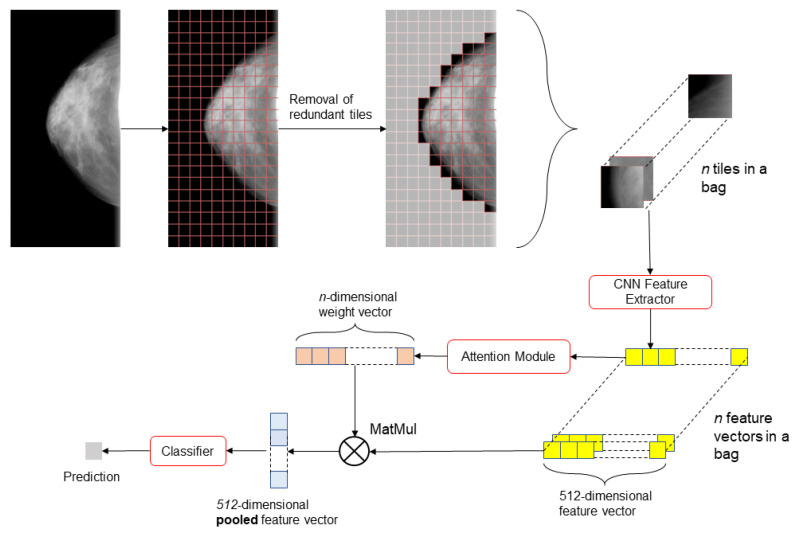
Outline of our approach to risk prediction using MIL and attention. The image is split into smaller, equally-sized sections, fed into a CNN-based feature extractor, and aggregated using attention.

Figure 3 describes the full end-to-end procedure of our approach with attention pooling. A pretrained ResNet-18 [18] feature extractor is first applied to every instance in a bag associated with the images. This extractor was chosen due to striking a good balance between proven performance on image processing tasks and reduced computational requirements. The instances are then pooled using attention as proposed by Ilse et al. [19] which can automatically assign importance to different instances in a given bag. The principle of attention-based pooling is to find a weighted average of instances using fully connected nets and pool based on the new averages. Based on experiments described by Ilse et al. [19,20] we have elected to use gated-attention pooling. This method is defined in Equations (1) and (2):

(1) $$z=\sum_{n=1}^{N}a_{n}x_{n}$$

(2) $$a_{n}=\frac{\exp\{w^{T}\tanh\left(Vx_{n}^{T}\right)⊙\mathrm{sigm}\left(Ux_{n}^{T}\right)\}}{\sum_{j=1}^{N}\exp\left\{w^{T}\tanh\left(Vx_{j}^{T}\right)⊙\mathrm{sigm}\left(Ux_{n}^{T}\right)\right\}}$$

where $\sum_{n=1}^{N}a_{n}=1$ and *w*, *V* and *U* are parameters of the network. Softmax ensures the weights add up to 1 to resemble the weighted average. Finally, the pooled feature vector *z* is fed into a linear perceptron to make the final prediction.

### 2.4. Experimental Setup and Model Evaluation

Experiments were conducted by 5-fold cross-validation on the 5064 women in the training data, with 15% percent of the training split used for validation. Folds were split per woman but images were inferenced individually. The outputs for the CC and MLO images per woman were averaged. Results from the PRC-mixed set are obtained using cross-validation. For the BCR-priors set, all of the produced models from cross-validation were ensembled and averaged as this set remained unseen throughout training. We used a learning rate of 0.000007 and an image-level batch size of 1, trained for 25 epochs. The validation set was used for early stopping and model selection. Negative log-likelihood is used as the loss function along with the Adam optimiser [21].

The model is evaluated using the Area Under the receiver operating characteristic Curve (AUC). Additionally, conditional logistic regression analysis was performed to adjust for known clinical breast cancer risk factors and give a better comparison against established clinical breast cancer risk models. Different cancer groups were also compared to evaluate how well performance was maintained across time.

## 3. Results

### 3.1. Logistic Regression Analysis

The deep learning model’s raw probability output was analysed by fitting a logistic regression model to predict cancer development. Since our data is case-control matched, a conditional model was used accounting for the age, BMI, parity, menopausal status, and HRT covariates. Additional, independent predictors not accounted for by the matching were also fitted: family history (first-degree relative, second-degree relative, both or neither), ethnic origin (self-reported), and alcohol use (yes/no). The intuition is that these factors would typically be considered by clinicians when determining an individual’s overall risk of breast cancer.

We compare MAI-risk output against two other tools that are typically used to measure risk. These include the averaged Visual Analogue Scales (VAS) as read by two experienced radiologists independently, and the IBIS risk scores as determined at the time of screening.

**Figure 4:**
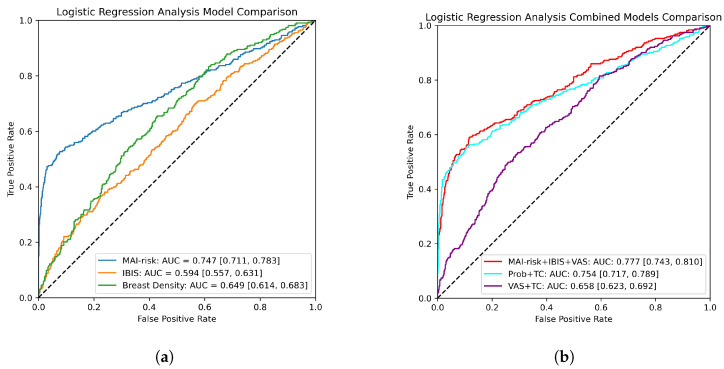
ROC curves comparing models. Discrimination is presented via AUC and bootstrapped to provide confidence intervals at 95%. (**a**) ROC curves comparing MAI-risk versus IBIS risk model and VAS. (**b**) ROC curves comparing combined models with VAS and IBIS risk model.

As can be seen from Figure 4a, MAI-risk significantly outperforms both the IBIS risk model and pure VAS in the task of risk prediction achieving statistical significance with an AUC of 0.747 (0.711, 0.783). Furthermore, our model’s curve begins with a high gradient which levels out as the False Positive (FP) rate increases. This is advantageous for our purposes as it suggests we can pick a threshold around the True Positive (TP) rate of 0.5 and effectively detect half of all high-risk women with minimal increase to the FP rate. At the higher end of the curve, the VAS model starts to become statistically superior.

The combined models are presented in Figure 4b. The incorporation of MAI-risk output exhibits the characteristic sharp gradient at the lower end, however, the addition of the other factors also improves the TP rate at the higher end. The best model is achieved when incorporating all three factors into a unified model, however, this model fails to attain statistical significance against our method alone.

Table 2 displays the odds ratios (OR) for the combined model (MAI-risk+IBIS+VAS) to demonstrate a comparison between the covariates. Whilst our model has the strongest OR at 3.04 (2.58, 3.59) comparatively, and achieves statistical significance, the fact the other factors have an OR above 1 suggests that they are also contributing to the decision-making process. For the IBIS model, this is anticipated as this model incorporates factors that cannot be presented in a mammogram such as detailed family history, however, the fact that VAS provides meaningful input suggests that our model does not fully capture risk associated with breast density to the same degree as VAS.

### 3.2. Model Performance for Different Sets

**Figure 5:**
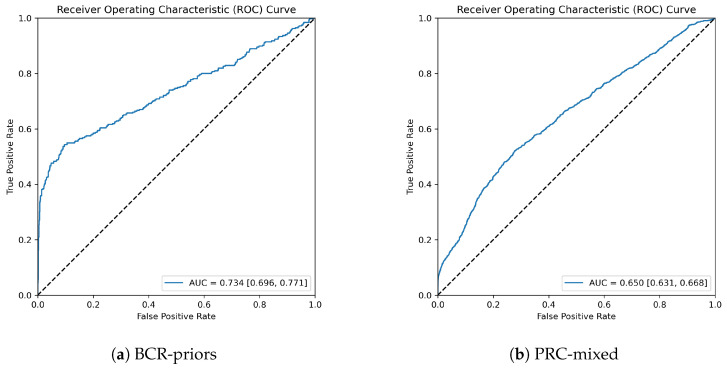
ROC curves for the two datasets. AUC is obtained by bootstrapping at 95% confidence.

Given that MAI-risk used cross-validation on the PRC-mixed set, we are able to demonstrate our model’s performance on this specific dataset and investigate how the model behaves in relation to different cancer groups. ROC curves can be seen in Figure 5 comparing results from the PRC-mixed set and the BCR-priors set.

Given the different testing procedures employed for the two sets, they cannot be compared directly. We can see that the results for PRC-mixed are not only statistically worse, but they have a more even curve gradient scaling. This may be attributed to the lack of ensembling for this dataset, or the nature of the BCR-priors set having cases that eventually turn to cancers sooner hence being easier to discriminate.

**Figure 6:**
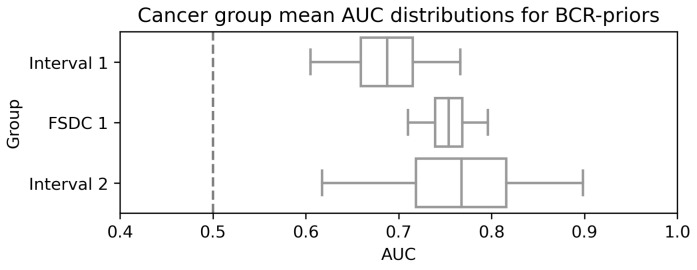
Figure displaying the AUC for the different cancer groups and their confidence intervals at 95% for the BCR-priors set.

**Figure 7:**
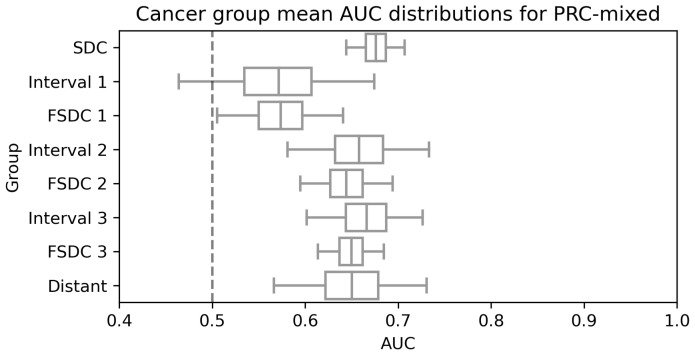
Figure displaying the AUC for the different cancer groups and their confidence intervals at 95% for the PRC-mixed set.

Stratifying by cancer group also allows us to examine the model performance by cancer group, a surrogate for time to diagnosis. Table 3 and its visualisation in Figure 6 display the results. It is clear that model performance does not decay as the cancer diagnosis becomes more distant from the time the analysed mammogram was taken. Interestingly, there is a significant reduction for earlier cancer groups, particularly those in Interval 1, but analysing results from BCR-priors in Figure 7, which constitutes of cases from those exact groups, we can see that the model has strong predictive power in these scenarios.

## 4. Discussion

This paper proposes a novel approach to image-based breast cancer risk prediction with a particular focus on short-term risk called MAI-risk. As demonstrated by the ROC curves in the previous section, the model has strong predictive power at lower thresholds without penalising the false positive rate making it particularly useful in a clinical setting. Since the model was originally conceived to be used alongside general population breast screening programmes, even a small increase to the FP rate can lead to unmanageable burden on national screening. However, due to the shape of our ROC curves, MAI-risk is able to alleviate the impact of this issue. Women who are suspected of developing cancer in the near future may be invited for additional screening sooner paving the way for personalised screening.

Logistic regression analysis has shown that whilst MAI-risk outperforms both VAS and IBIS in their short-term predictive power, both of these factors enhance the results in a combined model, which suggests that these factors are not fully captured by MAI-risk. For IBIS, this is a natural conclusion as it features factors inaccessible to an image-based model such as family history but is a more significant conclusion in terms of VAS. The combination of these details suggests that our model captures a broader range of image-based features that correlate with breast cancer risk but does not capture the effect of VAS to the same degree as two experienced radiologists. Thus, a noteworthy limitation of our approach is highlighted, wherein by only considering small patches at a time, we lose the global features of the breast. Nevertheless, our work emphasises the need for more targeted risk prediction tools that focus on mammographic features as opposed to solely relying on clinical risk factors and breast density volume.

Analysis of the AUC against cancer groups has demonstrated the model’s ability to discriminate between the formation of future cancers regardless of the time interval between the analysed mammogram and the diagnosis. Given the composition of the datasets, one might expect that mammograms that currently present a cancerous lesion may be easier to discriminate. However, this has not been the case. Isolated performance for the SDC group failed to achieve statistical superiority against the prior mammograms of cancers that form outside of the short-term, such as those in groups FSDC 2 and FSDC 3. These instances feature time intervals of up to 9 years between the cancer-free mammogram that was used for prediction and eventual cancer development. This highlights our models ability to combine aspects of early detection and true risk prediction whilst reinforcing the idea of using a mixed dataset for model development.

### Limitations

As discussed above, whilst the patching procedure permits us to utilise our risk prediction method at full resolution, it also hampers the model’s ability to look at global features. This primarily presented itself in the fact that our model was not able to fully capture VAS which is a total-breast area kind of feature and may be difficult to calculate from a collection of disjointed patches. This may also impact mammographic features that are too large to fit on a single patch. Additionally, the precise location of particular features is also lost. For example, breast lesions are most likely to develop in the upper outer quadrant [22], but due to the patching procedure, our model is not able to exploit this information. Future work would look to solve this issue by fusing global features alongside localised patches. The goal is to enable analysis of small features within a mammogram alongside a general overview of the breast simultaneously.

Another limitation can be seen when attempting to apply MAI-risk on alternative mammography image providers. MAI-risk has been wholly developed using General Electric Healthcare (GE) images and in its current state not suitable to be used with other image providers. This is a common issue in the deep learning field as AI models tend to be sensitive to domain shifts, such as a different image provider, and this would typically be resolved by retraining the model using an alternative dataset. This however may pose a challenge, as other providers may output images with a higher native dimension than GE which may make it difficult to maintain computational viability if the same approach is to be used. We may need to make adjustments to our approach to preserve the idea of prediction at full resolution if MAI-risk is to be adapted to other mammography providers.

## 5. Conclusions

MAI-risk is able to discriminate between cancer-free mammograms of women who go on to develop breast cancer up to 12 years into the future from women who remain cancer-free. We outperform two methods currently used to determine risk, IBIS and VAS, achieving statistical significance. MAI-risk is underpinned by the ability to make these predictions at full resolution without losing any fine detail and a dataset that has been case-control matched on common breast cancer risk factors eliminating most potential confounders that may introduce bias and lead to redundant models. The improvement of our model against both VAS and IBIS highlights the need for more image-based breast cancer risk models that can leverage the information contained within mammograms for the accurate stratification of risk groups.

## Figures and Tables

**Table 1:** Table displaying the varying level of patch overlap applied based on the total number of breast tissue pixels in an unpatched mammogram.

Number of Pixels Corresponding to Breast Tissue	Overlap Step Size
>5,017,600	200
2,508,800–5,017,600	168
752,640–2,508,800	112
<752,640	74

**Table 2:** Odds ratios calculated from logistic regression coefficients for the combined model (MAI-risk+IBIS+VAS). Normalised ratios are provided to ensure a fair comparison between the covariates.

Target Covariate	OR (Normalised)	OR (Representative)
VAS	1.60 (1.37, 1.88)	1.19 (1.05, 1.36)
IBIS	1.34 (1.08, 1.65)	1.03 (1.02, 1.04)
MAI-risk	3.04 (2.58, 3.59)	4.70 (3.74, 5.93)

**Table 3:** AUC breakdowns for the PRC-mixed set against cancer groups at 95% confidence.

Group	Number of Women	AUC
SDC	1600	0.676 (0.645, 0.707)
Interval 1	160	0.571 (0.464, 0.679)
FSDC 1	396	0.573 (0.506, 0.641)
Interval 2	268	0.657 (0.582, 0.730)
FSDC 2	700	0.644 (0.594, 0.693)
Interval 3	380	0.666 (0.602, 0.727)
FSDC 3	1312	0.650 (0.614, 0.685)
Distant	248	0.649 (0.565, 0.731)

## Data Availability

Data are contained within the article.

## Abbreviations

The following abbreviations are used in this manuscript:

MAI-risk	Manchester Artificial Intelligence risk
PROCAS	Predicting Risk of Cancer At Screening
PRC-mixed	PROCAS Remaining Cancers - mixed
BCR-priors	Breast Cancer Research - priors
SDC	Screen-Detected Cancer
FSDC	Future Screen-Detected Cancer
MIL	Mulitple Instance Learning
CNN	Convolutional Neural Network
FFDM	Full-Field Digital Mammography
BMI	Body Mass Index
HRT	Hormone Replacement Therapy
CC	CranioCaudal
MLO	MedioLateral Oblique
AUC	Area Under the receiver operating characteristic Curve
FP	False Positive
TP	True Positive
OR	Odds Ratio
GE	General Electric

## Appendix A

Table A1 outlines the detailed composition for both of the datasets used in our study. For the most part, the overall demographics are similar with key differences primarily associated with age. Menopausal status and HRT use are additionally impacted since both of these factors are highly dependant on age as well. These differences are explained by the design of these datasets, and how they relate to the larger PROCAS study. BCR-priors is an older dataset created whilst the PROCAS study was still ongoing with much more limited follow-up information, of around 5 years. PRC-mixed is a dataset that was conceived at a much later date and features more variable demographics due to possessing much longer follow-up information and a broader range of prior mammograms.

**Table A1:** Table showing the distributions of variables across the cases and controls for the two datasets. The data was self-reported by recruited women in a questionnaire at the time of entry. Family history was recorded in categories of first-degree relative only (FDR Only), second-degree relative only (SDR only), neither, and both.

		PRC-Mixed		BCR-Priors	
		**Cases (%)**	**Controls (%)**	**Cases (%)**	**Controls (%)**
Age	<50	210 (5.5)	129 (10.2)	16 (5.1)	47 (5.0)
	50–54	355 (28.0)	948 (25.0)	64 (20.3)	196 (20.7)
	55–59	245 (19.4)	1156 (30.4)	58 (18.4)	171 (18.0)
	60–64	303 (23.9)	504 (13.3)	99 (31.3)	297 (31.3)
	65–69	180 (14.2)	756 (19.9)	62 (19.6)	186 (19.6)
	>70	54 (4.3)	224 (5.9)	17 (5.4)	51 (5.4)
Menopausal Status	Premenopausal	165 (13.0)	500 (13.2)	22 (7.0)	67 (7.1)
	Perimenopausal	209 (16.5)	657 (17.3)	46 (14.6)	135 (14.2)
	Postmenopausal	835 (66.0)	2584 (68.0)	237 (75.0)	713 (75.2)
	Unknown	57 (4.5)	57 (1.5)	11 (3.5)	33 (3.5)
HRT Status	Current/Previous	470 (37.1)	1480 (39.0)	148 (46.8)	456 (48.1)
	Never	779 (61.5)	2316 (61.0)	166 (52.5)	485 (51.2)
	Unknown	17 (1.3)	2 (0.1)	2 (0.6)	7 (0.7)
BMI	<25 kg/m${}^{2}$	396 (31.3)	1193 (31.4)	99 (31.3)	292 (30.8)
	25–30 kg/m${}^{2}$	428 (33.8)	1283 (33.8)	111 (35.1)	337 (35.5)
	>30 kg/m${}^{2}$	341 (26.9)	1026 (27.0)	88 (27.8)	272 (28.7)
	Unknown	101 (8.0)	296 (7.8)	18 (5.7)	47 (5.0)
Ethnic Origin	White	1148 (90.7)	3500 (92.2)	282 (89.2)	866 (91.4)
	Other/Unknown	118 (9.3)	298 (7.8)	34 (10.8)	82 (8.6)
Year of Entry	2009	3 (0.2)	23 (0.6)	0 (0.0)	0 (0.0)
	2010	261 (20.6)	1267 (33.4)	63 (19.9)	178 (18.8)
	2011	449 (35.5)	1353 (35.6)	163 (51.6)	511 (53.9)
	2012	375 (29.6)	810 (21.3)	85 (26.9)	258 (27.2)
	2013	85 (6.7)	168 (4.4)	3 (0.9)	1 (0.1)
	2014	78 (6.2)	135 (3.6)	2 (0.6)	0 (0.0)
	2015	15 (1.2)	42 (1.1)	0 (0.0)	0 (0.0)
Family History	FDR Only	163 (12.9)	403 (10.6)	40 (12.7)	103 (10.9)
	SDR Only	216 (17.1)	606 (16.0)	51 (16.1)	153 (16.1)
	Both	82 (6.5)	163 (4.3)	23 (7.3)	46 (4.9)
	Neither	805 (63.6)	2626 (69.1)	202 (63.9)	646 (68.1)
Alcohol Use	Yes	961 (75.9)	2677 (70.5)	229 (72.5)	673 (71.0)
	No	281 (22.2)	1060 (27.9)	85 (26.9)	262 (27.6)
	Unknown	24 (1.9)	61 (1.6)	2 (0.6)	13 (1.4)
Parity	Yes	1085 (85.7)	3261 (85.9)	269 (85.1)	852 (89.9)
	No	179 (14.1)	537 (14.1)	44 (13.9)	95 (10.0)
	Unknown	2 (0.2)	0 (0.0)	3 (0.9)	1 (0.1)
